# The social status adversity and health in daily life moments study: ecological momentary assessment and ambulatory health assessments to examine meaning and mechanisms

**DOI:** 10.1186/s40359-024-01903-6

**Published:** 2024-07-19

**Authors:** Nataria T. Joseph, Laurel M. Peterson

**Affiliations:** 1 Department of Psychology, Pepperdine University, 24255 Pacific Coast Hwy, Malibu, CA 90263 USA; 2 Department of Psychology, Bryn Mawr College, Bryn Mawr, PA USA

**Keywords:** Socioeconomic status, Social stressors, Resilience, Ecological momentary assessment, Ambulatory blood pressure, Health behavior

## Abstract

**Background:**

African Americans and those of lower socioeconomic status (SES) are at disproportionate risk for hypertension- and cardiovascular-disease-related mortality relative to their counterparts. Progress in reducing these disparities is slowed by the facts that these disparities are difficult to mitigate in older adults and early origins of these disparities are poorly understood. The Social Status Adversity and Health in Daily Life Moments Study aims to precisely understand the proximal cognitive-emotional mechanisms by which unique social exposures disproportionately impacting these populations influence blood pressure (BP) parameters early in the lifespan and determine which individuals are more at risk.

**Methods:**

The study uses ecological momentary assessment (EMA) and ambulatory blood pressure (ABP) monitoring to assess race- and SES-based factors as they manifest in daily life moments alongside simultaneously manifesting cognitive-emotional states and ABP. A sample of 270 healthy African Americans between the ages of 18 and 30 is being recruited to complete two periods of 2-day, 2-night hourly ABP monitoring alongside hourly EMA assessments of socioeconomic strain, unfair treatment, and neighborhood strain during the waking hours. ABP data will be used to calculate ecologically valid measures of BP reactivity, variability, and nocturnal dipping. Other measures include actigraphy equipment worn during the monitoring period and comprehensive assessment of behavioral and psychosocial risk and resilience factors. Multilevel and multiple linear regression analyses will examine which momentary social adversity exposures and cognitive-emotional reactions to these exposures are associated with worse BP parameters and for whom.

**Discussion:**

This is the first time that this research question is approached in this manner. The Social Status Adversity and Health in Daily Life Moments Study will identify the cognitive-emotional mechanisms by which the most impactful race- and SES-based exposures influence multiple BP parameters in African American emerging adults. Further, it will identify those most at risk for the health impacts of these exposures. Achievement of these aims will shape the field’s ability to develop novel interventions targeting reduction of these exposures and modification of reactions to these exposures as well as attend to those subpopulations most needing intervention within the African American emerging adult population.

## Background

African Americans and those of lower socioeconomic status tend to be at disproportionate risk for hypertension and cardiovascular diseases (CVDs) [1, 2], disparities that present significant public health problems given the morbidity and mortality associated with hypertension and CVDs [3]. The early origins of these disparities and the mechanisms driving them remain poorly understood. Social factors with the most promise for understanding these racial and SES disparities given disproportionate exposure in these groups include socioeconomic strain, unfair treatment, and neighborhood adversities [4, 5]. Key physiological markers of early hypertension disparities are early cardiovascular stress reactivity, nondipping nocturnal blood pressure (BP), and high short-term BP variability. Without knowledge of exactly how these social factors influence these early BP parameters in emerging adults, we cannot develop the most pointed early interventions. No studies do the work of simultaneously measuring and connecting these social factors, BP parameters, and downstream proximal cognitive-emotional states that directly and acutely change BP and that are modifiable with intervention.

Numerous rigorous cohort studies, some longitudinal and some nationally representative, suggest that individual and neighborhood-level biopsychosocial risk factors may play a role in these disparities, with unfair treatment, neighborhood disorder, and neighborhood poverty being critical risk factors [6–9]. There is a need for more studies to comprehensively explicate the cognitive-emotional mechanisms by which these exposures impact cardiovascular outcomes. Perceived social threat is one such possible mechanism. Being treated poorly by those in one’s environment and identifying with a negative neighborhood environment can threaten a person’s sense of self, security, and perceived social status. These social threats are associated with physiological dysregulation, including dysregulation of the sympathetic nervous system important for regulating BP [10]. Integrating the social self threat concept into the Reserve Capacity Model [11] can provide a framework for testing cognitive-emotional mechanisms by which low SES impacts early BP markers of risk. The Reserve Capacity Model outlines that low SES is associated with increased exposure to negative experiences and emotions and reduced opportunity for positive experiences and emotions, the combination of which reduces our capacity for coping with the vicissitudes of life, leading to behavioral and physiological dysregulation and overall poor health. Social self threat is not explicated within the model but can be conceptualized as one the negative experiences that low SES increases. The Reserve Capacity Model is a risk model and not explicate the sources of resilience that allow many coping with lower SES to flourish. The most powerful resilience factors for African American emerging adults have yet to be determined, with some indications that social support and positive neighborhood factors may be important factors in reducing hypertension disparities faced by African Americans based on nationally representative large cohort studies [12].

Many studies in this literature compare African Americans to those of other racial/ethnic backgrounds, making it difficult to make conclusions about the rich variety of experiences and exposures within African Americans and difficult to make comparisons between African Americans at various places in the socioeconomic spectrum. There is a pressing need to disentangle race and socioeconomic status as large studies suggest that socioeconomic disparities partially account for cardiovascular risk disparities African Americans face [2].

Further, only small number of studies comprehensively investigate early cardiovascular risk in African American emerging adults, despite the fact that the health disparities they face emerge early in life and the fact that this developmental stage might represent the last opportunity to precisely and powerfully modify the processes of risk [13]. Emerging adults undergo biological, emotional, behavioral, and social changes that may set the stage for later cardiovascular disease. Small sample sized-studies suggest that African American adolescents exhibit higher BP than white adolescents and that negative emotions may be even more powerfully associated with BP in African American adolescents and adolescents dealing with lower SES relative to white adolescents and those with higher SES [14]. It is unclear whether this is true during the emerging adulthood stage as well.

### Socioeconomic factors in the daily lives of African Americans

Large cohort or national studies suggest that, within the African American adult population, both adulthood SES and retroactively reported childhood SES are powerfully inversely associated with hypertension risk [15, 16]. However, these studies also report some nuance in this relationship, with those that achieved upper SES mobility not consistently being at lower risk and, in fact, those African American adults of higher SES sometimes being at higher risk for hypertension risk factors such as obesity [17]. Large cohort or national studies have not been able to explicitly study the mechanisms that explain the overall SES-hypertension association within African American adults or the more nuanced findings regarding social mobility. Ambulatory BP reactivity, BP variability, BP dipping, physical activity, and sleep are markers for hypertension risk but large studies are unable to measure these using objective or gold standard measures. Thus, there is a substantial gap in the literature with respect to understanding the association between SES-related factors and these markers of hypertension risk. One study found that lower SES was associated with less BP dipping in a large sample of African American adults [18], and a small study found that lower SES and African American race/ethnicity are associated with less BP dipping [19]. The mechanisms driving these associations were not investigated. One longitudinal study found that lower SES was associated with higher average BP variability over the course of childhood, adolescence, and emerging adulthood and that that this association partially accounted for the finding that African Americans exhibit higher BP variability than Whites [20]. Again, the mechanisms driving these associations were not investigated. Further, large cohort or national studies often assess childhood SES retroactively and therefore are unable to investigate the mechanisms by which childhood SES influence hypertension risk. Studies of socioeconomic influences on hypertension risk need to be conducted at earlier stages of the lifespan to document how the association between socioeconomic status and hypertension risk unfolds. Again, the Reserve Capacity Model with enhancements focused on social self threat and resilience could inform this type of work.

A large, longitudinal national study found that lower SES in emerging adulthood, prior to 30 years of age, was associated with higher systolic BP [6]. This study provides evidence that high BP is evident even at this young age, with the majority of this nationally representative sample exhibiting high BPs. In this study, financial strain was associated with higher systolic BP. This study sample consisted primarily of white women so investigators were unable to drill down into SES issues within the African American population. Nonetheless, it did demonstrate that African American emerging adults had higher BP than white emerging adults.

Small-sample sized laboratory studies in African American adolescents have attempted to drill down on how the association between socioeconomic status and hypertension risk unfolds in early life, assessing both individual and neighborhood level SES and various BP parameters. One study, for example, found that neighborhood income was associated with ABP in adolescence even beyond individual family income and was not mediated by momentary health behaviors [14]. A different study found that BP reactivity was influenced by a combination of neighborhood poverty, household income, and parental education [21]. However, BP reactivity was conceptualized in this study as reactivity to a competitive video game, which likely does not capture the true social threats that African American young adults face across the SES spectrum. Studies examining ecologically valid forms of BP reactivity are sorely needed. Further, there is still the need to understand “the why”. In other words, this study showed that SES was associated with BP reactivity of adolescents but did not explicitly illuminate why this association occurs.

### Resilience in the daily lives of African Americans

The literature on hypertension risk-related resilience among African Americans is very piecemeal and incomplete. SES is associated with some forms of resilience in African Americans. For example, in middle-aged African Americans, childhood SES and adulthood social support are linked, with those of lower childhood SES having lower daily life social support in adulthood [22]. Other forms of resilience are associated with wellbeing in African Americans and are labelled as cultural aspects [23]. For example, ethnic pride and spirituality are associated with better wellbeing among African Americans [24, 25]. And, finally, some forms of resilience have been directly linked with BP parameters in African Americans. For example, in adolescent African Americans, self-esteem and optimism are associated with more BP dipping [19]. Some of these resilience factors such as social support and spirituality are frequently used by African Americans to cope with social adversities like unfair treatment [26]. There is a need to comprehensively test all of these factors together to determine which actually buffer the BP of African American emerging adults from the damages of everyday social adversities.

### The use of EMA to identify mechanisms

Ecological momentary assessment (EMA) is a valid and reliable methodology that has incremental utility for pinpointing psychosocial patterns occurring in everyday life and understanding their associations with health outcomes. Broadly, EMA methodology involves collecting repeated measures of data about daily life experiences, behaviors, and other psychosocial parameters as they occur in their natural environment at prespecified.

time intervals using portable devices [27]. EMA is likely to shed additional light as studies have shown that EMA-assessed psychosocial factors are more strongly associated with cardiovascular outcomes than global reports of psychosocial factors [28, 29]. EMA allows us to examine cognitive-emotional reactions (i.e., fear and appraisals of threat or harm) to momentary unfair treatment, socioeconomic strain, and neighborhood strain as they unfold, as well as vigilance and attentional processes focused on preparing for future perceived threats in these areas.

### BP nondipping, reactivity, and variability as indicators of later CVD risk

Beginning in late adolescence and young adulthood, African Americans and those of lower socioeconomic status have more adverse profiles on BP nondipping, reactivity, and variability [19, 30–32], all of which are associated with later hypertension, stroke, and/or other subclinical or clinical cardiovascular disease even when controlling for traditional risk factors in normotensive healthy individuals [33–41]. Increased daytime and nighttime short-term BP variability, although not well understood, seems to be impacted by the sympathetic nervous system, rigidity in blood vessels, sleep, and behavioral and psychosocial factors [36].

Given the importance of BP nocturnal nondipping, reactivity, and variability in predicting later cardiovascular disparities in healthy individuals, it is critical to determine modifiable correlates of BP nocturnal nondipping, reactivity, and variability in African American emerging adults. To the best of our knowledge, these three parameters have not been examined simultaneously in any project on healthy emerging adults. There is evidence of some linkage between variability, dipping, and reactivity, but the exact nature of this link amongst African American emerging adults has not been tested simultaneously in one study, so we do not know if they are wholly linked at this age in this population. With this project, we will be able to capture the comprehensive impact of social factors on the sympathetic nervous system that manifests in various ways including BP variability, reactivity, and nondipping.

### The current study

#### Aims

The study uses ecological momentary assessment (EMA) to assess race- and socioeconomic-based social factors as they manifest in daily life alongside simultaneously manifesting cognitive-emotional states and ABP parameters. Assessment of these factors will allow for clearer identification of social adversity’s impact on early hypertension risk. The project specifically aims to: (1) implement a new way of explaining how social adversity impacts hypertension disparities through examining associations among intra-individual momentary race- and SES-based experiences, momentary cognitive emotional reactions to them, and simultaneous ABP; (2) investigate between-individual factors that influence momentary cardiovascular reactivity to race- and SES-based experiences; and (3) investigate between-individual factors that influence nocturnal BP dipping and short-term BP variability in a novel manner, i.e., by collapsing across momentary experiences occurring within the same time window as dipping and variability parameters.

#### Conceptual & methodological innovation

Previous studies assess individual SES using relatively static income, education, and occupational status measures and neighborhood SES using relatively static poverty, income, and education measures. However, SES is not static. Further, the ways in which one’s SES influences daily life experiences is not static. SES directly influences our daily life financial strain, social interactions, neighborhood exposures, and self-evaluations. However, no studies examine the extent to which momentary fluctuations in these daily life SES factors influence momentary fluctuations in hypertension risk factors such as BP, physical activity, and sleep and simultaneously explores explanatory factors for these associations. For example, perceived threat and negative emotions are often linked to BP [42]. However, no studies explore these are mediators in momentary associations between daily life SES factors and ABP, physical activity, and sleep among socioeconomically diverse African American emerging adults. We assess all of these factors on an hourly basis using ecological momentary assessment (EMA), ambulatory blood pressure (ABP), and actigraphy in a sample of socioeconomically diverse African American emerging adults. This will allow us to distill global, distal psychosocial factors linked to early ABP parameters down to the basic, proximal exposures and cognitive-emotional states that are more directly connected to BP physiology as it is unfolding.

Further, previous studies explore the prominent influences of racial/ethnic identity and racial/ethnic unfair treatment on ABP, but none explore the impacts that socioeconomic identity and socioeconomic unfair treatment could have independent of racial/ethnic identity and unfair treatment although it is clear that various aspects of social identity threat or social evaluation are strong health predictors.

Finally, no studies of African American emerging adults explore BP reactivity, variability, and dipping together. This is important to do as, although all these BP parameters are influenced by the sympathetic nervous system, they may also reflect additional distinct physiological processes or some might be more heavily influenced by psychosocial factors than others. Relatedly, BP reactivity is usually assessed in an experimental environment whereas examine it outside of the laboratory. The combination of ABP monitoring and EMA will allow us to capture a snapshot of each participant’s BP reactivity tendencies as they go about their everyday lives, thereby maintaining higher ecological validity compared to experimental studies of BP reactivity. There appears to be some inconsistent correspondence between laboratory stress reactivity and ambulatory stress reactivity, again suggesting that EMA has incremental utility in this area [43].

## Methods and study design

The project methodology consists of anthropometric measures and questionnaires completed in two lab visits and four days of hourly EMA and ABP monitoring occurring between those two lab visits.

### Participants

We aim to recruit a healthy sample of 270 African Americans between the ages of 18 and 30 and with access to a mobile phone. We aim to recruit an equal number of males and females although, based on previous data collections using similar methodology in this population, we expect that we will recruit more females than males. Primary exclusion criteria include pregnancy (women); a history of hypertension or antihypertensive medication use, cardiovascular incidents, or diabetes; a history of major mental illness (schizophrenia, bipolar disorder, PTSD); current night shift work; diagnosed sleep disorders; and use of medication known to affect the cardiovascular system or sleep. Participants are recruited using physical and electronic public methods (i.e., Craigslist posts, social networking ads, flier postings, and postings to online newspapers/websites geared towards the African American Community) as well as community-targeted methods. The community-targeted methods involve identifying key community “gatekeepers” and institutions such as churches and barbershops that are willing to distribute and post fliers.

### Procedures

Participants arrive at the research office to have anthropometric measures taken by research staff, complete questionnaires, and complete training on the field monitoring procedures. During the office visit, research staff first assess each person’s weight and height with shoes removed. Then, research staff assess clinic BP according to established guidelines from the American Heart Association and the European Society of Hypertension [44]. Participants are instructed to walk 100 feet from the lab through the hallway and back. They then sit down quietly for five minutes with their legs uncrossed. Next, their clinic BP is taken twice, with a one minute pause in the interim. Research staff then measure participants’ arm circumferences to fit them with the most appropriate ABP cuffs and calibrate the ABP monitor by conducting 2 readings separated by a two minute rest period and calculating the difference between the readings. Staff consider calibration complete if there is not a large difference between the two readings or switch cuffs if there is a large difference between the two readings. Research staff then program the ABP monitor to inflate hourly. During the field monitoring training, participants are instructed to complete EMA questionnaires on their phones immediately after each automatic BP reading completed by their ABP monitors. The next day, they begin field monitoring, which consists of two days of monitoring, a one day break from monitoring, and two additional days of monitoring. Each day of field monitoring includes a questionnaire completed upon awakening, hourly ABP monitoring hourly and EMA questionnaire completion, and a questionnaire completed prior to bedtime. After field monitoring is complete, participants return to the research office to complete a questionnaire querying the extent to which various psychosocial and behavioral factors unfolded during the field monitoring period and to return the equipment.

### Materials

#### Demographic and self-report health measures

Age, gender, marital status, and working status are assessed using standard self-report questions. Family history of hypertension and cardiovascular disease are assessed using five items querying family history of hypertension, hypertension prior to age 60, heart disease, heart attack, and stroke. Instructions specify that, for the purpose of these items, family includes only parents and siblings.

#### Anthropometric measures

Research staff use a Seca 703s medical scale to measure assess height and weight. BMI will be calculated using the standard formula (lbs/inches^2^ × 703). Research staff use a Seca 203 ergonomic circumference measuring tape with extra WHR calculator to measure each participant’s waist circumference, hip circumference, and WHR.

#### BP assessment

##### Clinic BP

A Dinamap BP monitor is used to assess clinic BP. The average of two BP readings is the clinic BP measure for each participant.

##### ABP

ABP is assessed using the SunTech Oscar2™, which has been validated according to several internationally recognized protocols [45, 46]. The Oscar2TM uses the auscultatory method of assessing BP. AccuWin Pro Software will be used to consolidate and clean ABP data. For each participant, BP reactivity will be calculated in multiple stages. BP reactivity will be calculated using only BP readings assessed while the participant is awake and completing EMA assessments. For reactivity calculations, participants’ BP readings will be matched and merged with their EMA data collected within 5 min of that reading; anything outside of that window will be discarded as it is not conceptually aligned with acute reactivity. Each participant’s reactivity scores will be estimated using a multilevel regression modeling the effect of each respective social adversity factor on ABP as a random effect. For each participant, nocturnal BP dipping will also be calculated in multiple stages. First, each participant’s average nighttime BP and average daytime BP will be calculated separately for systolic and diastolic BP, and then average nighttime BP will be divided by average daytime BP to create that participant’s nocturnal dipping score, with higher scores representing less dipping. Nighttime BP will be every reading that occurs in the sleep window determined by each participant’s actigraphy data and checked with that participant’s self-report sleep and wake times. All other readings will be considered as daytime BP. Finally, for each participant, the standard deviation of BP readings will be calculated to assess BP variability. Daytime and nighttime BP variability will be calculated separately.

##### ABP covariates

Important ABP covariates are assessed during hourly EMA questionnaires and include location, posture, temperature, talking, and physical movement.

#### SES measures

##### Global SES measures

Objective SES is assessed using self-reported education and yearly household income as well as Census-derived education and income measures from self-reported zip codes. Specifically, participants are instructed to self-report the highest grade and the highest degree they completed (personal education) and the highest grade and degree each parent completed (parental education). Further, they are instructed to select the range in which their annual pre-tax household incomes falls, with twelve range options representing $5,000 and $10,000 intervals until $75,000, at which point the remaining interval is “$75,000 or more”. Given the development stage of the participants under study, participants are instructed to include all sources of income in their household income, including any money or financial help they receive from family members. Participants self report zip codes. Zip codes are used to assess neighborhood SES. Specifically, the freely available US Census data [47] provides information on the percentage of people in each zip code that are living in poverty and that achieved particular levels of education as well as median and mean household income for each zip code.

Subjective SES is assessed using the United States and community versions of the MacArthur Scale of Subjective Social Status [48]. This scale consists of pictorial representations of 10 rung ladders and instructions to click on the rung that best represents the participant’s perceptions of where he or she stands relative to others in the United Status (US version of the ladder) and relative to others in his or her community (community version of the ladder). Instructions include elaboration that those with more education, income, and highly respected jobs are at the top of the ladder and that those with less of these resources are at the bottom of the ladder.

SES stress is assessed using the 12-item Economic Hardship Index [49]. Example items include “Have you had trouble buying food or other necessities for your family?” and “Has your landlord ever threatened to evict you because you could not pay your rent?”. Participants reply “yes” or “no” to indicate whether each of these has occurred within the past year.

##### EMA SES measures

SES stress is assessed using the following two items measured on a 6-point scale: “My financial situation is strained” and “I am concerned about my financial situation”.

#### Social identity and unfair treatment measures

##### Global social identity and unfair treatment measures

Black identity is assessed using the Multidimensional Inventory of Black Identity (MIBI; [50]). Socioeconomic identity is assessed using a modified version of the MIBI, e.g., “Overall, *my socioeconomic status* has very little to do with how I feel about myself” instead of “Overall, *being Black* has very little to do with how I feel about myself”.

Meta-perceptions regarding SES identity are assessed using three items querying to which SES group participants believe others classify them and the extent to which participants believe that their SES identity is apparent to others. Items include “to what extent do you think others are able to guess your socioeconomic status just by looking at you [talking to you]?” and “to which socioeconomic status do you think others think you belong?”.

Race-based unfair treatment is assessed using Detroit Area Study Everyday Unfair Treatment Scale [51]. SES-based unfair treatment is assessed using a modified version of the Detroit Area Study Everyday Unfair Treatment Scale, e.g., “Because of your *SES*, you are treated with less respect than other people” instead of “Because of your *race*, you are treated with less respect than other people”.

##### EMA social identity and unfair treatment measures

EMA-adapted items from the MIBI, meta-perceptions, and Detroit Area Study Everyday Unfair Treatment Scale used for global assessments were used for momentary assessments.

#### Neighborhood adversity measures

##### Global neighborhood adversity measures

Neighborhood disorder is assessed using the 7-item scale from the panel study called the Project on Human Development in Chicago Neighborhoods. Neighborhood violence is assessed using the 7-item scale used by others [52].

##### EMA neighborhood adversity measures

Three items adapted from the global measures assess whether the participant saw signs of neighborhood disorder or violence in the last hour.

#### EMA cognitive-emotional social threat measure

Five EMA items assess the extent to which a person feels threatened, not secure, and vigilant for problems on a 6-point scale.

#### EMA resilience measures

As we and others have done in past research, items from previously established scales were adapted for EMA. EMA-adapted items query self-esteem [53], spirituality and religiosity [54], control [55], autonomy [56], social support and belonging [57], and ethnic and socioeconomic pride [50]. Data reduction of these items will occur prior to analysis. Specifically, these items will be totaled together to create one resilience score for each moment of each participant’s data. Our previous EMA research in a different African American emerging adult sample using most of these items has demonstrated that these items are manifestations of the same core latent resilience construct [58]. We will conduct a maximum likelihood factor analysis to confirm that these items manifest from a unitary construct in this sample as well. Other work has used most of these items together as an EMA scale as well [59].

#### Perceived stress and emotion

Perceived stress is assessed on the global level using the 14-item Perceived Stress Scale (PSS; [60]) and at the hourly EMA level during the monitoring period using a two-item EMA-adapted version of the PSS. Positive and negative emotions are assessed at the global level using the 20-item Positive and Negative Affect Scale (PANAS; [61]) and at the hourly EMA level using a 7-item EMA-adapted version of the PANAS.

#### Objective and self-report behavioral measures

An Actigraph GT9X-BT watch is used to objectively assess sleep and physical activity behaviors. Actigraphy has been validated against polysomnography (Crespo, Fernández, Aboy, & Mojón, 2013). ActiLife 6 software will be used to consolidate and clean sleep and physical activity data. Self-report physical activity is assessed using the International Physical Activity Questionnaire (IPAQ; [62]) and self-report sleep is assessed using the Pittsburgh Sleep Quality Index (PSQI; [63]).

#### Beginning of day (BOD) and end of day (EOD) questionnaires

These questionnaires assess self-report time to bed and time of awakening as well as morning and overall daily reports of financial stress, social identification, unfair treatment, neighborhood disorder and violence, perceived stress, emotion, perceived cognitive-emotional social threat, and resilience that use the same items as the hourly EMA questionnaires, except participants are instructed to assess these with the day rather than the past hour as the time frame of reference.

### Statistical plan

All analyses will be conducted using SPSS. The MIXED procedure will be used to conduct multilevel regressions to investigate within-persons hypotheses and the REGRESSION procedure will be used to conduct multiple linear regressions to investigate the between-persons hypotheses. G*Power [64] was used to determine sample sizes needed for adequate power for between-persons analyses, resulting in a determination that adequate power (at least 80%) would be present in a sample of 263 participants for small effect sizes (Cohen’s f^2^ = 0.05 based on *R*^2^ of 0.05). Monte Carlo stimulations using MLPowSim [65, 66] determined power for within-persons regressions, resulting in a determination that adequate power (at least 80%) would be present if approximately half of the 263 participants needed for between-persons analyses provided at least 48 data points each (approximately 80% EMA-ABP compliance from each participant). Amount and sources of missing data will be diagnosed. The SPSS Missing Data module will be used to determine whether data is missing at random. Depending on several factors, we will consider using a multiple imputation using a chained equations (MICE) program [67] or the multiple imputation procedure in SPSS.

All analyses will control for demographic factors, BMI, smoking status, and past month drinking behavior. Given sex differences in various psychosocial factors and all BP parameters used as outcomes in analyses [68], each analysis will be first run in the overall sample and then run separately in males and females in sex-stratified analyses.

In multilevel regressions testing within-persons hypotheses that ABP is higher during moments of social adversity, i.e., socioeconomic stress, SES- or race-based unfair treatment, and neighborhood problem exposure, we will allow the intercepts to be random but keep the coefficients fixed, and use autoregressive covariance structures and the restricted maximum likelihood estimation method. The basic model for these regressions will include the person-mean centered momentary social adversity variables, participant means for each momentary social adversity variable, and covariates, including momentary ABP covariates. Person-mean centered momentary values and participant mean values for each momentary social adversity variable are included to allow the model to truly distinguish between between-persons and within-persons associations. The full model will also include momentary negative cognitive-emotional states of fear, perceived harm, perceived threat, attentional focus to threat, and vigilance for future threat to test the hypothesis that these factors mediate the momentary associations between the social adversity exposures of interest and the ABP. If the relationships between the momentary social adversities and ABP diminish substantially in the full model, this will provide evidence that momentary negative cognitive-emotional states mediate these associations. We will then use the indirect test macro for multilevel models developed by Dr. Daniel Bauer to further confirm this mediation [69]. Separate analyses will be run on systolic ABP and diastolic ABP.

With respect to multiple linear regressions testing between-persons hypotheses that BP reactivity to social adversity is lower in those exhibiting resilience factors, reactivity coefficients created for each person will be the outcome and mean level of momentary resilience will be the predictor variable. Separate analyses will be run on systolic ABP reactivity and diastolic ABP reactivity. If mean momentary resilience factor is significantly associated with ABP reactivity, we will run exploratory analyses to identify which dimensions of the resilience factor are more powerfully associated with ABP reactivity.

With respect to multiple linear regressions testing between-persons hypotheses that nocturnal BP dipping and BP variability are lower in those with higher mean socioeconomic stress levels, higher mean SES- or race-based unfair treatment levels, higher mean exposure to neighborhood problems, and higher mean negative cognitive-emotional states, separate analyses will be run on systolic nocturnal BP dipping, diastolic nocturnal BP dipping, systolic BP variability, and diastolic BP variability.

## Discussion

The Social Status Adversity and Health in Daily Life Moments Study uses a complex set of procedures and rich materials to test mechanistic between-persons and within-persons hypotheses regarding the processes that unfold early in life to set the stage for SES-based and race-based disparities in hypertension and cardiovascular risk. This project will identify the mechanisms by which the most impactful race- and SES-based exposures influence multiple BP parameters as well as resilience factors that reduce the health impacts of these exposures. The long-term objective of the study is to contribute to the understanding and subsequent reduction of SES and race-based disparities in cardiovascular risk by identifying key modifiable risk processes in emerging adults. This is an important endeavor given that behavioral, emotional, and social risk processes are more difficult to mitigate in older adults [70].

## Data Availability

No datasets were generated or analysed during the current study.

## Abbreviations

ABP: Ambulatory blood pressure
BP: Blood pressure
BMI: Body mass index
EMA: Ecological momentary assessment
SES: Socioeconomic status
WHR: Waist-to-hip ratio
